# In Situ TEM Observation of Cooperative Grain Rotations and the Bauschinger Effect in Nanocrystalline Palladium

**DOI:** 10.3390/nano11020432

**Published:** 2021-02-09

**Authors:** Ankush Kashiwar, Horst Hahn, Christian Kübel

**Affiliations:** 1 Institute of Nanotechnology, Karlsruhe Institute of Technology, Hermann-von-Helmholtz Platz 1, 76344 Eggenstein-Leopoldshafen, Germany; ankush.kashiwar@partner.kit.edu (A.K.); horst.hahn@kit.edu (H.H.); 2 Department of Materials and Earth Sciences, KIT-TUD Joint Research Laboratory Nanomaterials, Technische Universität Darmstadt, Alarich-Weiss-Straße 2, 64287 Darmstadt, Germany; 3 Electron Microscopy for Materials Science (EMAT), University of Antwerp, Groenenborgerlaan 171, B-2020 Antwerp, Belgium; 4 Institute of Mechanics, Materials and Civil Engineering, Université Catholique de Louvain, Place Sainte Barbe 2, B-1348 Louvain-la-Neuve, Belgium; 5 Karlsruhe Nano Micro Facility (KNMF), Karlsruhe Institute of Technology, Hermann-von-Helmholtz Platz 1, 76344 Eggenstein-Leopoldshafen, Germany

**Keywords:** nanocrystalline metals, nanomechanical behavior, plasticity, thin films, Bauschinger effect, deformation mechanisms, grain rotation, in situ transmission electron microscopy (TEM), automated crystal orientation mapping in STEM (ACOM-STEM)

## Abstract

We report on cooperative grain rotation accompanied by a strong Bauschinger effect in nanocrystalline (nc) palladium thin film. A thin film of nc Pd was subjected to cyclic loading–unloading using in situ TEM nanomechanics, and the evolving microstructural characteristics were investigated with ADF-STEM imaging and quantitative ACOM-STEM analysis. ADF-STEM imaging revealed a partially reversible rotation of nanosized grains with a strong out-of-plane component during cyclic loading–unloading experiments. Sets of neighboring grains were shown to rotate cooperatively, one after the other, with increasing/decreasing strain. ACOM-STEM in conjunction with these experiments provided information on the crystallographic orientation of the rotating grains at different strain levels. Local Nye tensor analysis showed significantly different geometrically necessary dislocation (GND) density evolution within grains in close proximity, confirming a locally heterogeneous deformation response. The GND density analysis revealed the formation of dislocation pile-ups at grain boundaries (GBs), indicating the generation of back stresses during unloading. A statistical analysis of the orientation changes of individual grains showed the rotation of most grains without global texture development, which fits to both dislocation- and GB sliding-based mechanisms. Overall, our quantitative in situ experimental approach explores the roles of these different deformation mechanisms operating in nanocrystalline metals during cyclic loading.

## 1. Introduction

In coarse-grained (cg) metals, the rotation of grains during plastic deformation is dominated by their orientation with respect to the straining direction, and the resulting texture can be predicted well by classical plasticity models, such as Taylor and Sachs models or self-consistent approaches [1,2,3,4,5]. The grain rotation in cg metals is often accomplished by a microscopic dislocation glide on multiple active slip systems leading to texture formation [6]. The first statistically significant measurements of a large number of individual micron-sized grains rotating during the deformation of a bulk specimen were reported by Margulies et al. using 3D X-ray characterization [7]. They compared their experimental results with classical predictions. Comparison with the Taylor model showed significant discrepancies around the <100> corner and in the middle of the stereographic triangle, whereas self-consistent approaches predicted large variations of the rotation behavior in the <100> corner but did not predict the experimentally observed rotation directions [2,7]. The Taylor model is based on the assumption that each grain experiences the same strain as the surrounding bulk material, equal to the macroscopic plastic strain, and accommodates the strain via the five independent slip systems, which are required to describe the state of strain in face-centered cubic (FCC) metals [3,5,8,9]. The validity of the critical resolved shear stress for simultaneous activation of these five independent slip systems at the same external stress state and the equilibrium of the stress state has been questioned [10]. Alternatively, the Sachs model assumes a uniform external state of stress for each individual grain and predicts crystallographic slip only for those slip systems where the critical resolved shear stresses are approached according to Schmid’s law [5]. However, the Sachs model does not include constraints for shape changes induced by the activation of different slip systems in differently oriented grains and, therefore, does not guarantee a shape equilibrium between the deforming grains and their neighbors [5,11]. Various modified models have been introduced in the past decades to account for interactions between single grains and all their neighbors (assumed as a matrix regardless of the different orientations of each grain) or the detailed interactions between neighboring grains [10,11,12,13,14,15,16]. So far, these studies have been mostly confined to cg metals and alloys with grain sizes of several (tens of) micrometers, where dislocation-based slip is the dominant deformation mode driving the grain rotation and ultimately giving rise to the texture formation.

For grain sizes in the nanoscale regime, below 100 nm, multiple concurrent deformation mechanisms have been proposed, both dislocation and grain boundary (GB) mediated [17,18,19,20,21,22,23,24], and the complex interplay between these is far from being fully understood. The texture evolution observed experimentally during deformation in nanocrystalline (nc) metals has been shown to be strongly affected by the different types of deformation mechanisms, and thus, texture analysis is considered a potential approach to untangle dislocation and GB-mediated plasticity in nc metals [25]. It is generally accepted that the GB-mediated rotation of grains does not lead to a global texture development [6,20,25]. For example, Ivanisenko et al. reported GB-mediated processes controlling the deformation in nc palladium for grain sizes below 40 nm [26]. It was observed that the intensities of the characteristic shear texture components were several times stronger in cg metals than in their nc counterparts, which was considered as evidence of GB-mediated deformation in nc metals [20,26]. Using in situ experiments in a scanning electron microscope and electron backscattering diffraction, collective grain rotation was shown in nc metals and was attributed to a mechanism that accommodates the strain by the rotation of grains as rigid units [27]. In contrast, Kobler et al. experimentally confirmed the importance of twinning and detwinning during the straining of nc palladium–gold thin films [28] and dislocation processes contributing to the deformation [25,29]. Furthermore, Yang et al. suggested dislocation-based plasticity as a dominant mechanism even at grain sizes as low as 28 nm and were able to describe the texture development by classical models limiting the number of slip systems [21].

Another indication of the role of dislocation activity in nc materials stems from the Bauschinger effect (BE), which has been attributed to microstructural heterogeneity leading to dislocation pile-up at the GBs and interfaces and their release during relaxation [30] and was discovered experimentally in the last two decades for thin films [31,32]. Using in situ TEM testing, Mompiou et al. showed clear experimental evidence of partial re-emission of dislocations from the GBs and their reverse motion during loading/unloading in tensile-deformed ultrafine-grained (UFG) Al to elucidate on the unusually large inelastic reverse deformation upon unloading, which has not been reported in the case of the cg counterparts [33]. Recently, Izadi et al. reported on the BE in UFG Al and showed that the inelastic strain recovery is associated with the partial or completely reversible rotations of the grains during unloading [34]. Furthermore, Mompiou et al. reported on grain rotation in UFG Al and showed that rotations occur as a collective process involving several neighboring grains and are a direct consequence of GB dislocations with the Burgers vector out of the plane of the film [35].

For a full understanding of the deformation behavior of nc materials, a direct link between the relevant deformation mechanisms and both local and global microstructural characteristics, like grain size distribution, crystal orientation, GB character, and texture, needs to be established. Ex situ mechanical testing methods provide precise control over load, displacement, and temperature and are extensively employed to measure the mechanical response of nc metals. However, ex situ tests only enable correlation with the microstructure on an averaged statistical basis by characterizing the initial and selected deformed states, which further ignores relaxation effects during unloading. Although ex situ testing and characterization have yielded valuable insights into the deformation mechanisms of nc metals, in situ mechanical testing techniques are needed for an analysis of the deformation dynamics without contributions from relaxation processes and sample-to-sample variations. In situ nanomechanics enables capturing the real-time microstructural changes for each specimen and simultaneous measurements of the macroscopic mechanical response during deformation for a direct correlation. In situ nanomechanical testing inside an electron microscope can further provide high spatial resolution with reasonable statistics for reliable quantification of the grain structure, size distribution, crystallographic orientation, and various other microstructural parameters in order to experimentally follow the complex grain interactions and the nature of their deformation both individually and for a statistically meaningful ensemble. These locally resolved in situ measurements allow for the direct visualization and analysis of the deformation processes occurring at individual grains or clusters of grains. Since grain rotation in nc metals is highly sensitive to the operating deformation mechanisms, a systematic combination of local and statistically meaningful investigations on grain structure changes can improve our understanding of the dominant deformation mechanisms contributing to the collective plastic deformation through direct correlation of the microstructure and the macroscopic mechanical properties. The in situ experimental approach can shed light upon the interplay of the active deformation mechanisms at the nanoscale that control the texture evolution and can enable refinement of the current theories of plasticity with improved fidelity for this class of materials. Here, we are using a combination of in situ nanomechanical testing in TEM and automated crystal orientation mapping (ACOM) in scanning transmission electron microscopy (STEM) mode to experimentally follow the rotation of a large number of individual grains in nc Pd to elucidate the underlying active deformation mechanisms, including the role of the Bauschinger effect during relaxation. Our experimental approach is a step towards a direct experimental visualization and quantitative understanding of the complex interaction between grains in nc metals during mechanical deformation, particularly focusing on dislocation pile-up and grain rotation.

## 2. Experimental: Material Synthesis, Methods, Data Acquisition, and Processing

### 2.1. Specimen Preparation for In Situ TEM Nanomechanics

**Figure 1:**
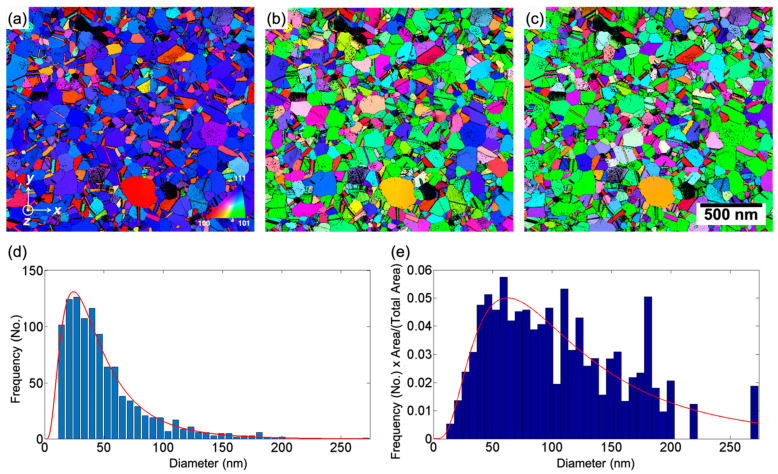
Inverse pole figure color maps showing the crystallographic orientations along the (**a**) *z*-, (**b**) *x*-, and (**c**) *y*-axes according to the coordinate system shown in (**a**), (**d**) number and (**e**) area weighted grain size distribution.

A 60 nm thick nc Pd film was deposited using radio frequency magnetron sputtering on a carbon-coated TEM substrate (Quantifoil holycarbon R2/1 + 2 nm C, Quantifoil Micro Tools GmbH, Germany). The film deposition was achieved by five cycles of 50 s. Each cycle was interrupted for 10 s. The chamber was maintained at a base pressure of 10^−8^ mbar. A sputter pressure of 0.005 mbar and a sputter power of 60 W were employed. The as-sputtered film consists of very fine grains (~10 nm), leading to significant overlap due to projection through the thickness of the specimen. This would render the microstructural investigation by TEM, especially using quantitative ACOM-STEM, highly challenging. For a reliable quantitative TEM analysis, these projection effects need to be excluded. Therefore, the grain size was increased by annealing. It turned out that a shorter heat treatment immediately after sputtering did not lead to sufficient grain growth. However, aging in ambient environment for several months resulted in a film consisting of mainly a single layer of grains with minimum projection effects. After storing the sample for 18 months at room temperature, it was annealed at 350 °C for 10 min using a Gatan 652 heating holder (Gatan Inc., Pleasanton, CA, USA) inside a Tecnai F20 ST TEM (FEI Company, Hillsboro, OR, USA). The resulting microstructure after heat treatment exhibited a columnar growth with preferential {111} texture along the growth direction (Figure 1a). Within the plane of the sample, a weak {101} texture is visible as indicated by inverse pole figure color maps along the specimen *x*- and *y*-axes (Figure 1b,c). The bright-field TEM (BFTEM) images and the corresponding selected area diffraction patterns of the sample before and after in situ heat treatment in the TEM are shown in Figure S1a,b. The number and area weighted average size distributions are shown in Figure 1d,e. The number and area weighted grain size distribution (Figure 1d,e) is fitted with a log-normal distribution with modes of 25 and 63 nm. A sample for in situ straining was prepared on a push-to-pull (PTP) device (Bruker, Minneapolis, MN, USA) using a Strata 400S DualBeam (FEI Company, Hillsboro, OR, USA) focused ion beam (FIB) system following the procedure introduced in [29], as illustrated in Figure S2a–c.

### 2.2. In Situ TEM Nanomechanical Experiments

**Figure 2:**
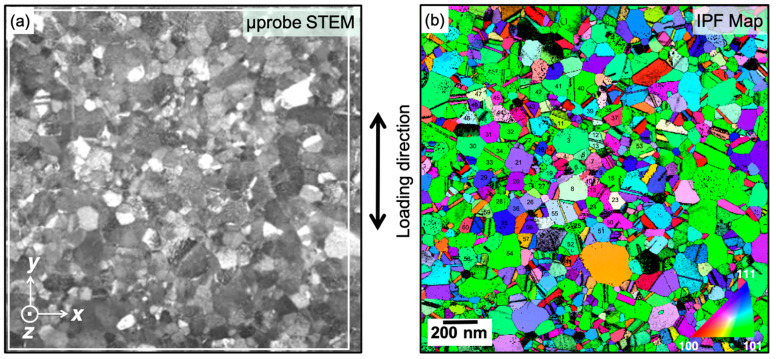
(**a**) μP-STEM image and (**b**) inverse pole figure (IPF) map along the loading direction acquired using ACOM-STEM. The grains analyzed are numbered as reference for further analysis.

**Figure 3:**
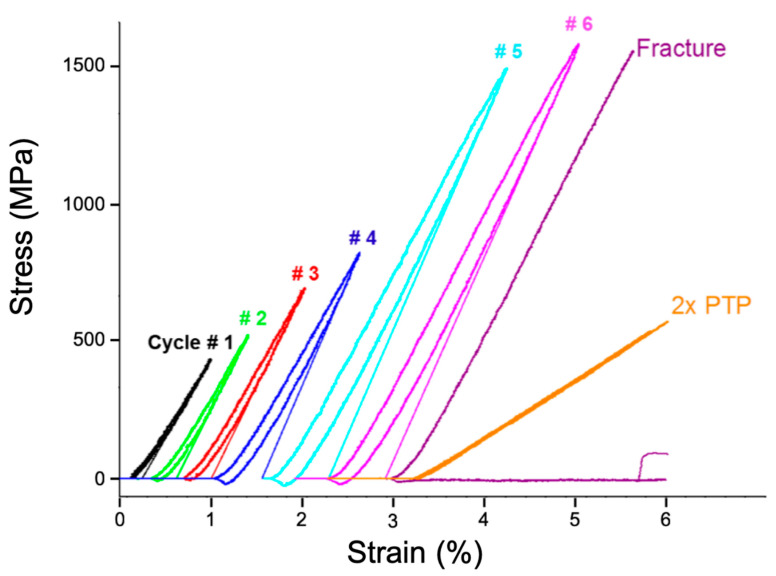
Stress–strain response during cyclic loading–unloading experiments after drift correction showing a significant Bauschinger effect. The curves in orange show the response of the push-to-pull device, which are fully overlapping during loading–unloading, indicating a full elastic response of the PTP device and the stability after drift correction.

The straining experiments were performed using a PI 95 TEM PicoIndenter (Bruker, Minneapolis, MN, USA). The sample was strained using displacement control and subjected to repetitive loading and unloading cycles at a displacement rate of 10 nm/s along the *y* image axis (Figure 2). The maximum displacement was increased progressively during each cycle. The in situ nanomechanical response during cyclic loading–unloading experiments was corrected for drift due to mechanical instabilities, vibrations, or thermal fluctuations. For a faithful drift correction over several loading–unloading cycles, any manual interaction with the PicoIndenter to adjust its position was avoided during the experiment. This ensured that any mechanical drift of the indenter remained almost constant through all the cycles that were recorded over time and was confirmed by an analysis of the time stamps when the indenter made contact with the PTP device. The recorded displacement was corrected by subtracting the measured drift using the corresponding time stamps at each data point. The fidelity of the drift correction was confirmed by the excellent match of the loading and unloading curves obtained for the PTP device after the fracture of the film, indicating a true elastic response of the PTP device. The PTP device’s contribution to the load was subtracted from the experimentally measured stress–strain curve of the sample on the PTP device, and the resulting stress–strain response representing only the sample is shown in Figure 3. Following the in situ TEM deformation experiments, straining series were recorded using load control in microprobe STEM (μP-STEM) mode. Precession electron diffraction-based ACOM-STEM orientation maps were acquired with a nominal beam diameter of about 1.5 nm (without precession), a pixel size of 4 nm, a semiconvergence angle of ~1 mrad, and a precession angle of 0.4° during holding segments at different loading levels using the ASTAR system (NanoMEGAS, Brussels, Belgium) following the approach reported by Kobler et al. [36]. The ACOM series was acquired for one complete cycle (loading and unloading) with a maximum strain of 6.3% and for reloading of up to 7.6% before the fractured sample. In addition, μP-STEM-ADF images were obtained with a camera length of 80 mm using the high-angle annular dark-field (HAADF) detector before and after each ACOM map. As a reference, a μP-STEM-ADF image and an ACOM map acquired at the undeformed state are shown in Figure 2a,b.

### 2.3. Quantitative Analysis of ACOM-STEM Data

The ACOM maps were indexed using the ASTAR software suite version V2.0 from NanoMEGAS [37,38,39]. The orientation refinement algorithm in the NanoMEGAS ASTAR software V2.0 was used to index the orientation maps. The algorithm developed by Rauch et al. improves the angular resolution by interpolating the diffraction intensities between successive orientation templates [40], resulting in greater accuracy of approximately 0.3° for the orientation determination. This was further confirmed by Leff et al. [41]. The resulting crystal orientation maps were processed for a reliable quantitative analysis of the grain rotation using a modified version of the procedure reported by Kobler et al. [36,42]. In order to exclude the bending or tilting of the specimen, evaluation of the global grain rotations was carried out with the undeformed state as reference. For the reliable analysis of the grain rotations, the grains were selected from the deformation stages in which the specimen did not show any noticeable bending. The quantitative analysis and interpretation of the grain rotations and the global texture was performed using MTEX 5.2.beta2 by Bachmann et al. [43]. The statistical analysis of the grain orientation and orientation changes is presented based on 55 grains representing ~25% of the imaged area as indicated in Figure 2b.

### 2.4. Nye Tensor Analysis of Geometrically Necessary Dislocations

Nye tensor analysis was performed to estimate the density of geometrically necessary dislocations (GNDs) using the ATEX software version 1.21 (Université de Lorraine, Metz, France) by Beausir and Fundenberger [44]. For the analysis of the GND density, a kernel orientation filter was applied with 1 neighboring pixel and a maximum exclusion angle of 5°. The maximum disorientation angle used for the analysis was 5°, and a smoothing level of 2 was applied for noise reduction. The maximum value of the entrywise norm of the Nye tensor was scaled to 3 μm^−1^ to obtain the spatial distribution of the scalar GND density as color maps.

## 3. Results and Discussion

### 3.1. In Situ Nanomechanical Response: The Bauschinger Effect

The in situ nanomechanical response during the initial stages of cyclic loading–unloading experiments is dominated by a pseudo-elastic deformation of the thin film as shown in Figure 3. At lower stress levels, the mechanical response is deviating slightly from the linear behavior in all cycles. This is presumably due to systematic artefacts related to slight unbending/bending of the sample and due to readjustment of the contact between the indenter and the PTP device. For each cycle with progressively increasing maximum strain, the stress–strain response exhibits a characteristic deviation from the linear elastic behavior during unloading, and the strain in the fully relaxed (unloaded) state shows a significant plastic recovery of 25–30% of the maximum applied strain. This is a clear indication of the Bauschinger effect (BE), which leads to plastic recovery during relaxation, and the effect is increasing with increasing strain. The BE seen here is similar to the results reported by Xiang et al., Rajagopalan et al., and Mompiou et al. [31,32,33]. As reported previously, the observed BE is indicative of strong stress buildup at the grain boundaries and/or the film–substrate interface due to dislocation pile-up [31,33,45,46]. The back stresses that develop due to dislocation pile-up lead to a reverse motion during unloading and result in a reverse plastic deformation as reported by Mompiou et al. for UFG materials [33]. However, during the loading part of each cycle, the stress–strain response in all cycles, including the one where the sample fractured, is almost linear with no pronounced observation of a sharp transition from a linear (pseudo) elastic regime towards the onset of plasticity. This is due to the fact that nc metals exhibit an extended microplastic regime [47,48]. In our case, only a slight deviation from the linear (pseudo) elastic deformation was observed close to the maximum strain in cycles 5 and 6.

### 3.2. Structural Observations and Deformation Characteristics of the Nanocrystalline Palladium Film

**Figure 4:**
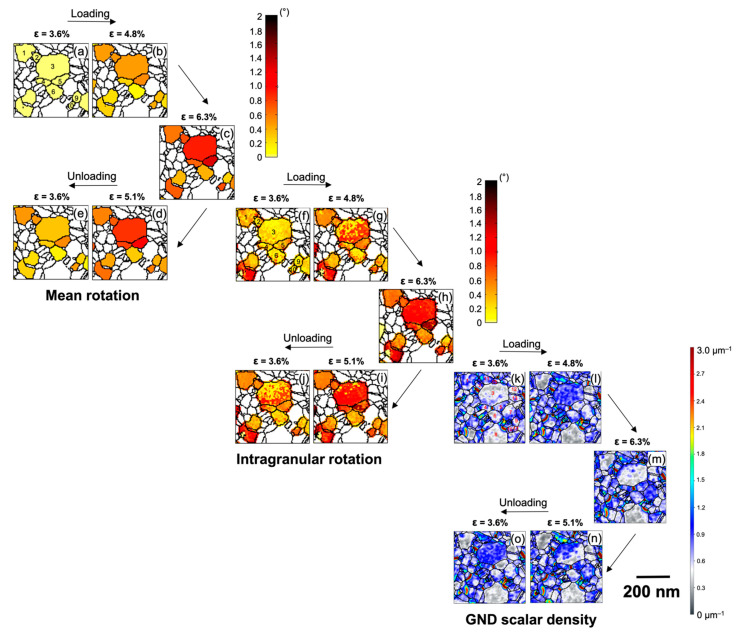
(**a**–**e**) Mean grain rotation maps and (**f**–**j**) intragranular rotation maps at the given strains; grains in (**a**) are numbered as in Figure 2b. The colored grains in each map are the ones analyzed with their rotation (in degrees) as indicated in the color bar; (**k**–**o**) scalar density maps of geometrically necessary dislocations expressed as entrywise norm of the Nye tensor.

**Figure 5:**
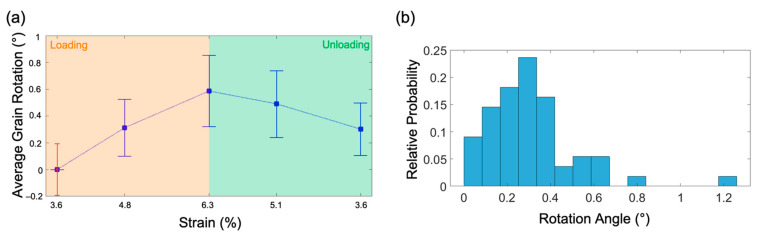
(**a**) Average grain rotation analyzed for 55 grains at different strain levels. The red error bar at 3.6% strain indicates the average of the standard deviation of the orientation variation in each individual grain, including variations due to curvature within the grain and small angle boundaries. The blue error bars indicate the standard deviation of the mean rotation angles at each deformation step. (**b**) Statistical analysis of the distribution of the rotation angles of grains during straining from 3.6% to 6.3%.

The nc Pd sample shows a wide distribution of grain sizes ranging from 13 to 200 nm (Figure 1d,e), with grains in the range of 20 to 150 nm diameter making up about 90% of the grains, reflecting a somewhat heterogeneous microstructure of the sample. We analyzed the rotation of a large number of grains distributed throughout the area imaged and noticed that the early stages of straining and last stages of unloading exhibit some bending/tilting of the film (Figure S3a). This is due to some residual plastic deformation (elongation) from earlier straining series recorded for this film to characterize the mechanical properties. From 3.6% strain to 6.3% and back to 3.6%, the grain rotation maps do not reveal any long-range orientation gradient (Figure S3b), showing that there is virtually no bending or tilting of the Pd film occurring during this part of the straining series. All states of the sample that showed noticeable bending were excluded from further analysis to avoid any misinterpretation by mixing the rotation of grains due to the mechanical deformation and rigid body tilting/bending of the overall film and to prevent misinterpretation because of changes in the projected grain structure [42]. Figure 4 shows grain rotation maps (evaluated as described in [29]) and compares the mean rotation (Figure 4a–e) of each grain and the rotation at the pixel level representing intragranular activity (Figure 4f–j). The colors indicate the degree of rotation relative to the average grain orientation at 3.6% strain used as a reference state for the entire analysis. While an absolute grain rotation of around 1° is small, it is still significant compared with the measurement accuracy estimated from the average of the standard deviation of orientation variations within each grain of 0.2° (Figure 5a). As the grains are sometimes bent and contain small angle boundaries, the true measurement accuracy for the average grain orientation is expected to be better than 0.2°.

Looking at the average rotation of each grain (Figure 4a–e), it can be seen that the grains exhibit a distinct rotation during loading due to the mechanical deformation of the sample (bending/tilting of the film is excluded). During unloading, a reduction of the rotation of the grains can be seen. This is a local example for the overall behavior depicted in Figure 5a, which shows the average deformation-induced grain rotation evaluated for 55 grains, which increases during loading and decreases by ~50% during unloading. Figure 5b shows the probability distribution of the rotation angles measured for the analyzed grains with the majority exhibiting grain rotations of over 0.2°. The distinct rotation, with variations in both magnitude and direction, of individual grains in close proximity to each other, as exemplified in Figure 4a–e, depicts a heterogeneous response to the deformation. This leads to an inhomogeneous stress distribution and buildup of back stresses at grain boundaries. The heterogeneity of the microstructure in terms of differences in grain size, grain orientation, grain boundary type, and orientation leads to this heterogeneous stress development, which is driving the BE in agreement with previous reports on the BE in thin metallic films [32,46,49]. This is also driving the observed backward rotation during unloading fitting to the mechanical response of the thin nc Pd film (Figure 3). Thus, the BE is, to some extent, accommodated by the partial reversibility of grain rotation during unloading.

**Figure 6:**
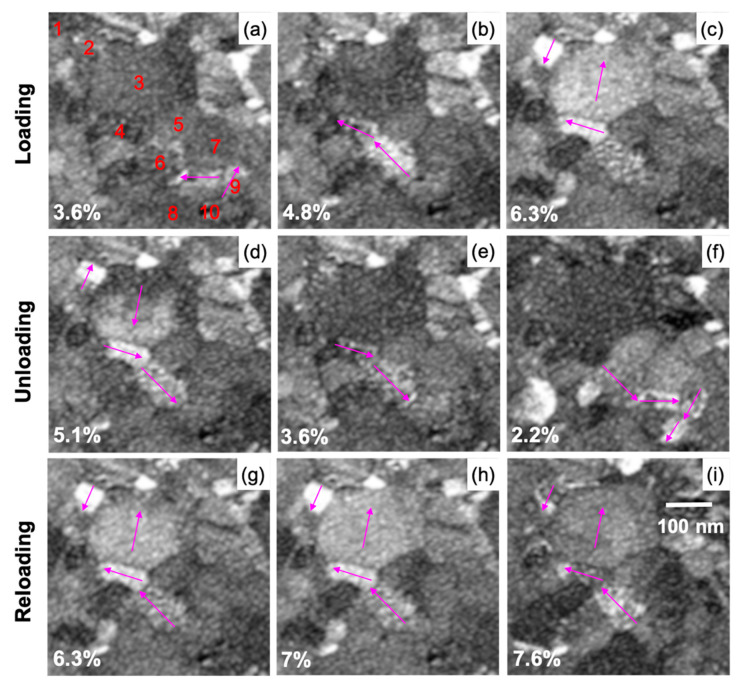
μp-STEM-ADF images acquired after each ACOM map acquisition showing cooperative rotation of grains: (**a**–**c**) loading, (**d**–**f**) unloading, and (**g**–**i**) reloading. The corresponding strain at every deformation state is indicated in the image. Grains in (**a**) are numbered as in Figure 2b.

The intragranular rotation maps (Figure 4f–j) reveal a significant rearrangement of subgrain boundaries as it is prominently observed in grains 1, 3, and 5, suggesting intra-granular dislocation activity to contribute to the deformation process. Some direct evidence of dislocation activity was obtained using in situ BFTEM imaging during the cyclic loading and unloading experiments. Movie S1 shows the forward and backward motions of a dislocation in a 100 nm wide grain as the specimen is loaded and unloaded, indicating some contribution of dislocation activity to the observed BE. This observation fits the results shown by Mompiou et al., who clearly demonstrated reversible dislocation motion in an ultrafine-grained aluminum sample in in situ BFTEM experiments for somewhat larger grains [33,50]. For randomly oriented small-grained materials, direct experimental evidence of reversible dislocation activity is difficult to obtain, but, for example, molecular dynamics simulations by Farkas et al. revealed the motion of small angle boundaries (dislocation arrays) in grains as small as 6 nm [51]. A detailed analysis of the dislocation activity seen in our in situ BFTEM series was not possible due to the small grain size and the random grain orientation. Nevertheless, the contrast changes observed in the in situ ADF-STEM series, which depends on the (out-of-plane) crystallographic orientation, are not uniform in individual grains (Figure 6 and Movie S2), further supporting intragranular dislocation activity with a noticeable out-of-plane component as one of the mechanisms behind the grain rotation similar to previous reports on UFG materials [23,29,52,53,54].

For a more quantitative analysis, we evaluated the geometrically necessary dislocation (GND) density to describe the local intragranular lattice curvature [55,56] observed in the in situ ACOM-STEM series. This approach ignores the contribution of pairs/sets of statistically stored dislocations (SSD) that do not lead to a net orientation change within the ~5 nm spatial resolution of the ACOM-STEM maps. We analyzed the Nye dislocation density tensor [55] using an approach that was implemented in the past for electron backscatter diffraction [57], as well as ACOM-TEM data [41,58]. The GND scalar density (*ρ_GND_*) can be expressed as the entrywise norm of the Nye dislocation density tensor (*α*) divided by the length of the Burgers vector (*b*):

(1) $$\rho_{GND}=\frac{1}{b}\sqrt{\alpha_{ij}\alpha_{ij}},$$

where *α_ij_* corresponds to the curvature components of the dislocation density tensor. For 2D mapping, five curvature components of the Nye dislocation density tensor are obtained as described by Pantleon as [56]: *α*_12_, *α*_13_, *α*_21_, *α*_23_, and *α*_33_. From these five components, the truncated scalar GND density ($\rho_{GND}^{2D}$) is computed as [57,59]:

(2) $$\rho_{GND}^{2D}=\frac{1}{b}\sqrt{\alpha_{12}^{2}+\alpha_{13}^{2}+\alpha_{21}^{2}+\alpha_{23}^{2}+\alpha_{33}^{2}},$$

Figure 4k–o shows the spatial distribution of $\rho_{GND}^{2D}$ represented as color maps at varying strain levels during loading and unloading. Considerable changes in GND density can be seen in some of the grains (e.g., grains 3, 5, 7, and 11), whereas others barely show any significant change in GND density (e.g., grains 1, 8, 9, 12, and 13). This is another evidence of the strongly inhomogeneous response of the grain structure to the deformation, leading to heterogeneous stress development at the boundaries. Looking at grains 5 and 7, the overall GND density increases during loading (Figure 4m) and reduces during unloading (Figure 4o). In contrast, the neighboring larger grain 3 shows complex changes in GND density both during loading and during unloading, whereas grain 11 shows an almost continuous increase in GND density during loading and further during unloading to ε = 5.1% (Figure 4n). It is worth noting that the GND density in grain 11 is mostly concentrated in the vicinity of its GBs. In particular, the density at the boundary to grains 3 and 15 increases during unloading to ε = 5.1% (Figure 4n). This sharp rise in GND density reflects the development of dislocation pile-ups in grain 11 in the vicinity of the boundaries with grains 3 and 15 during unloading from ε = 6.3% to ε = 5.1%. Interestingly, this pile-up at the GBs almost disappears as the specimen is further unloaded to ε = 3.6% (Figure 4o), whereas a high GND density is developing through grain 3, suggesting that the dislocation activity initiated in grain 3 from the boundary with grain 11. Thus, dislocations contribute to the intragranular plasticity at least for those grains where the GND density is observed to evolve significantly, and these dislocations, to some extent, contribute to the observed rotation of some of the grains (Figure 4a–j). The evolution of GND density is significantly different for some sets of grains in close vicinity to each other, indicating that these grains are subjected to significantly heterogeneous stress fields within the length scales of few tens of nanometers. There is a significant generation of back stresses during the unloading of the specimen as it can be seen from an increase in GND density within some grains and especially at the GBs. The development of these dislocation pile-ups at the GBs and the back stresses eventually contribute to the observed BE as discussed in Section 3.1.

**Figure 7:**
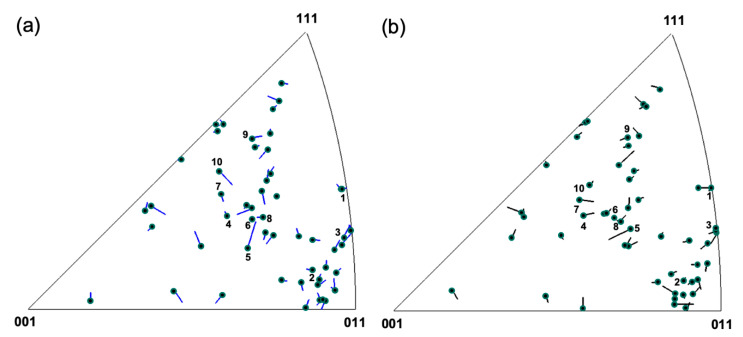
Inverse pole figures showing the final position of the loading axis of the grains labeled in Figure 2 together with vectors magnified four times representing the orientation change: (**a**) during loading from 3.6% to 4.8% strain and (**b**) during unloading from 5.1% to 3.6% strain. Vectors corresponding to grains 1 to 10 as in Figure 2b have been annotated.

The analysis of the grain rotation and GND density evolution in Figure 4 illustrates heterogeneous stress development and the contribution of dislocation activity to the deformation process. With the strong back stresses present at the GBs, this raises the question to what extent the grain rotation in this nc specimen can be described considering the grain orientation and the globally applied stress as it is typically done in cg materials. Therefore, we analyzed the orientation dependence and directionality of the rotation of a large number of grains with respect to the straining direction to extend the local analysis shown for a few grains in Figure 4 towards a statistically meaningful ensemble analysis considering the grains throughout the film. Figure 7 shows a vector representation of the rotation of the tensile axis for each grain in an inverse pole figure (IPF) during loading and unloading. It clearly shows that the rotation of the tensile axis for each grain is along different directions, and often grains with similar crystallographic orientation show different rotation directions. This is despite the fact that the applied strain is small and should therefore only activate the primary slip systems, which should result in a strong orientation dependence of the grain rotation, if a pure dislocation-based mechanism without interaction between the grains is considered. Nevertheless, in most cases, the vectors are pointing in exactly opposite direction during loading and unloading, indicating a reversible rotation of the grains, which is in agreement with the observed BE. Winther et al. comprehensively analyzed the lattice rotation of individual grains in a bulk material with grain sizes of a few microns [1,2,60]. While there were some noticeable deviations from the Taylor and Sachs models, they were able to experimentally show that the overall grain rotation strongly depends on the grain orientation and primarily attributed these to the slip-based processes [2]. The deviations were explained by the interaction of grains and stress development at the GBs even for this large grain size. Similar interactions were further reported in [10,12,13,16]. Thus, even a dislocation-based mechanism can lead to a randomization of texture if significant interactions and stress buildup between grains are present (e.g., as seen in a strong BE).

Despite the observed contribution of dislocations to the deformation process discussed above, some small grains, such as 1, 6, 9, and 10, rotate uniformly without noticeable change in the subgrain boundary structure (Figure 4f–j). In agreement with this observation, the GND density (Figure 4k–o) within these grains does not seem to change significantly during deformation. This may be the result of dislocations that have been emitted and escaped at another grain boundary or free surfaces between the recorded straining states. Therefore, it is not possible to detect the complete dislocation activity and subgrain boundary evolution experimentally. However, another explanation is the contribution of GB-mediated processes, which lead to a rotation of nanoscaled grains as rigid units with minimal or no dislocation activity involved, leaving the intragranular structure unaltered. This has been suggested, for example, by Shan et al., who observed the rotation of small grains (~10 nm) and explained that they occur in conjunction with GB sliding (GBS) [23]. Ivanisenko et al. explained their experimental results for Pd even with a mean grain size of 130 nm with a strong contribution of GBS [27]. In a theoretical study on GBS, Swygenhoven et al. assumed no dislocation contribution to the plasticity from the grain interior to explain the deformation [61]. In such a grain-boundary-dominated process, the rotation of the grains is not governed by classical dislocation slip leading to the formation of crystallographic texture, but the rotation is considered “non-crystallographic” in nature. The driving force for the rotation originates from the misorientation dependence of the GB energy and the relationship with all grains in their neighborhood [62,63,64]. Thus, this mechanism involves grain rotation coupled with GBS and necessitates the accommodation of the resulting strain and strain rate incompatibility between clusters of neighboring grains [27,65]. Gutkin et al. explained a crossover from GB sliding to rotational deformation in a plastic flow by the splitting of gliding GB dislocations into climbing GB dislocations at triple junctions [66]. These climbing GB dislocations constitute walls that move along GBs adjacent to the triple junction, thereby causing a lattice rotation within the grain.

Apart from grain rotation, GB sliding is often reported to occur coupled with GB motion or diffusion through GBs or grain interiors [62,63,64,67]. Simulations by Swygenhoven et al. suggest atomic shuffling and stress-assisted migration of free volume to be accommodated by hopping processes involving several GB atoms [67]. However, since our in situ experiments were performed at room temperature (0.16 T_m_ of the homologous melting temperature of Pd), diffusion-based processes are not expected to be dominant in our deformation experiments. Nevertheless, with our experimental approach focusing on ACOM-STEM, we cannot detect these processes, and they may contribute to some extent to the deformation in our sample.

Based on the discussion of dislocation-based processes in this nc film, our analysis emphasizes that a lack of texture development does not necessarily guarantee the deformation to be dominated by GB-mediated processes as it is commonly accepted for nc metals.

### 3.3. Cooperative Grain Rotation (CGR)

Figure 6 shows μp-STEM images of a selected set of grains (same region as shown in Figure 4) during a loading, unloading, and reloading cycle with the corresponding strain levels indicated in the images. The purple arrows indicate how the contrast changes propagate from one neighboring grain to the next. The corresponding movie of the straining series is available as supplementary information (Movie S2) for better visibility of the evolution of the contrast changes. As shown in Figure 6a–c, the contrast changes propagate from grains 6 to 4 to 3 during loading and reverse direction during unloading, propagating from grains 3 to 4 to 6 towards 9 and 10 (Figure 6d–f). On reloading (Figure 6g–i), similar contrast changes are observed as during the initial loading. The μp-STEM-ADF images are sensitive to changes in the diffraction conditions and thus the crystallographic orientation of the grains. Therefore, these contrast changes are an indication of grains showing a cooperative change in orientation (grain rotation) as ACOM-STEM measurements were used to exclude bending/tilting of the sample as discussed in the previous section. Considering the annular geometry of the HAADF detector, the observed contrast changes are due to grains rotating with a strong out-of-plane component, whereas pure in-plane rotations are not observed under these conditions.

Based on the ACOM-STEM orientation determination in Figure 4a–e, Table S1 shows the Schmid factor analysis for grains 1 to 10 assuming a uniaxial stress tensor along the *y*-axis (global straining direction). Because of the strong {111} texture of the film, the majority of the grains have <110> {111} slip systems almost parallel to the plane of the film (or at a small inclination) and are referred to as in-plane slip systems, whereas the remaining 9 slip systems constitute out-of-plane slip systems. The in-plane slip systems have lower Schmid factors compared with the out-of-plane slip systems since the in-plane slip plane normals are nearly orthogonal to the loading axis. This results in a low resolved shear stress for the in-plane slip components. Together with the fact that the film is mostly composed of a single layer of grains throughout the thickness of the film, meaning that most grains have a free surface on the top, there is a high probability for the out-of-plane shear components to be activated. This explains the strong tendency of the grains to undergo out-of-plane rotation during deformation.

A variety of mechanisms for collective or simultaneous grain rotations in nc metals have been reported in the past using different TEM techniques, like dark-field TEM [23], HRTEM [68], and a combination of bright-field and ACOM-TEM [35]. Wang et al. observed a simultaneous (collective) rotation of several neighboring grains in a coordinated manner and explained it by the climbing of dislocations at triple junctions and grain boundaries, whereas no intragranular dislocation processes were observed [68]. These observations were made using HRTEM and were restricted to the analyses of in-plane rotations. In our study, combining microprobe STEM imaging and ACOM-STEM, we observed significant out-of-plane contributions.

As seen in Figure 4b–c and 4c–e, the rotation of grains 6 to 4 and 4 to 3 gradually increases the fitting to the STEM series in Figure 6 and Movie S2, which shows the contrast changes propagating in steps from one grain to the next at different deformation states. This is also analogously apparent during unloading and reloading. This indicates that the CGR is occurring in a stepwise fashion and at least partially driven by dislocation motion unlike the collective rigid body rotations of the grains observed (e.g., by Wang et al.) [68]. Overall, this confirms that localized plastic recovery via cooperative and significantly reversible rotation of grains is contributing to the BE.

**Figure 8:**
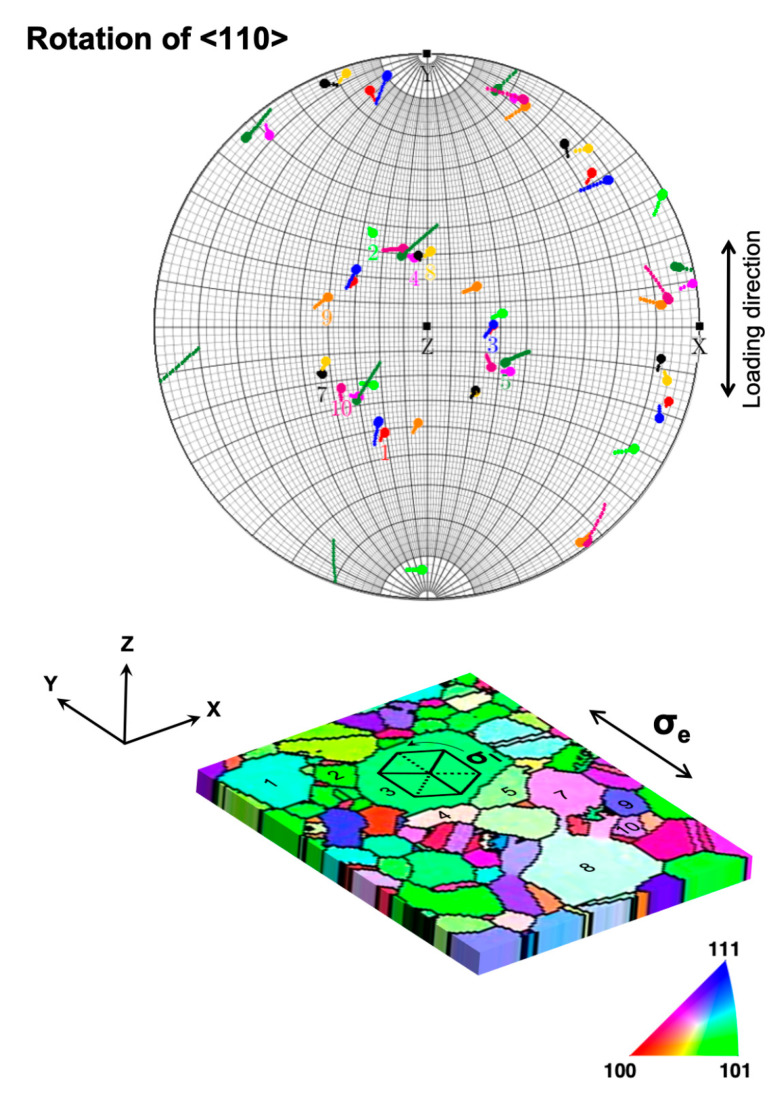
Stereographic projection showing the rotation of the slip directions for grains 1–10. Circular markers indicate the positions of the slip directions before and after deformation. The rotation of the slip direction is indicated by lines, with their length increased 15 times for visibility. A schematic of the sample reference frame is shown, where X–Y represents the plane of the film, and Z is the electron beam direction, with the spatial coordinates X, Y, and Z coinciding with those of the coordinates in the Euler space. The IPF color map is shown along Y. The sample is subjected to an external uniaxial stress σ_e_ along Y. A schematic of an FCC crystal with {111} orientation along Z is shown rotating under the influence of local stress field σ_l_ surrounding it.

Figure 8 shows all <110> slip directions of the grains analyzed in Figure 4a–j. The initial and final positions at ε = 3.6% and ε = 4.8% strains are highlighted in the pole figure by (overlapping) circular markers, and the rotation direction is indicated by a line, with the length enhanced by a factor of 15 for visibility. The in-plane slip systems all have a low Schmid factor and are presumably not activated so that in Figure 8, out-of-plane slip systems are most likely to be activated. An analysis of the rotation trajectories shows that the slip direction changes appear to be fairly randomly oriented. Some of the slip direction changes (e.g., grains 9 and 5) are oriented along the great circles parallel to the *y*-axis, indicating that these tend to rotate towards the global straining direction as would be expected for the deformation of single-crystal FCC metals. However, a significant number (e.g., grains 2, 3, 4, and 8) are oriented at a high angle to the great circle, in agreement with the strong local stress buildup at GBs and GB-dominated processes controlling their response as discussed in Section 3.2.

**Figure 9:**
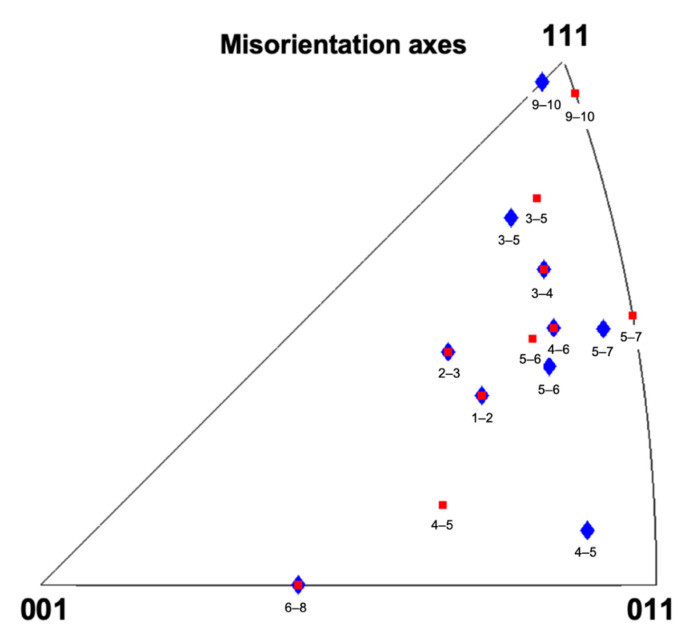
Misorientation axis represented in an IPF before and after deformation, denoted by blue and red markers.

A surprising feature observed for the grains indicated in Figure 6 (except for grain 5, which did not participate in the CGR) is that they seem to maintain the misorientation axis between the pairs of grains throughout the deformation (Table S2, Figure 9), which suggests that the grain boundary character is not strongly changing during the CGR.

**Figure 10:**
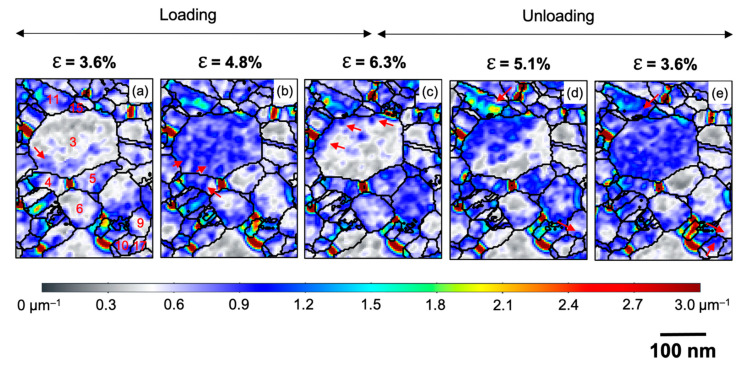
Illustration of cooperative grain rotations on the basis of nucleation, propagation, absorption, and piling up of dislocation at or in the vicinity of certain GBs marked using arrows. The corresponding strains at each stage during loading are (**a**) 3.6%, (**b**) 4.8%, (**c**) 6.3% whereas during unloading are (**d**) 5.1% and (**e**) 3.6%. The grains of interest are number in (**a**).

As seen in Figure 10a, the initial GND density within most parts of grain 3 is low, but the density is noticeably higher in close proximity to the GB with grain 4 (indicated by the arrow in Figure 10a), and the GNDs appear to extend inside grain 3. The GND density at the GBs of grain 3 and other neighboring grains is mostly lower. As the strain is increased to ε = 4.8% (Figure 10b), the GND density abruptly increases throughout the grain, while the density in proximity to other neighboring GBs is similar to the grain interior. These observations indicate that dislocations are preferentially nucleating from GB 3–4. The stress field at GB 3–4 is sufficiently high for dislocations to propagate from GB 3–4 to the interior of the grain. At ε = 4.8%, a high dislocation density is seen on both sides of GB 3–4 at the same time, indicating a continued rotation of grains 3 and 4, which is in agreement with the simultaneous contrast change in the STEM series (Figure 6b,c). On further straining to ε = 6.3%, the GND density within grain 3 is reduced, but some high GND density is seen in proximity to GBs with the neighboring grains (indicated by the arrows in Figure 10c). This indicates that dislocations propagated further and were absorbed at GBs at the other end of grain 3.

**Figure 11:**
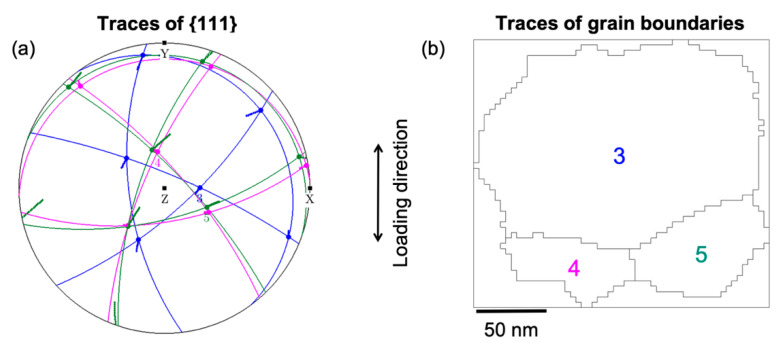
(**a**) Traces of {111} planes of grains 3, 4, and 5. Other marking conventions are the same as shown in Figure 8. (**b**) Grains 3, 4, and 5 with traces of their grain boundaries and the rotation axes and their sense of rotation.

The grain rotations analyzed in detail above not only depend on their grain orientation and size but are also significantly influenced by their neighbors and the geometric arrangement of the grain boundaries in agreement with the discussion in Section 3.2. In order to further elucidate the role of these factors, we analyzed the geometric relationship of the slip systems in grains 3, 4, and 5, where only grains 3 and 4 participate in the CGR. Figure 11a shows the traces of the {111} slip planes of grains 3, 4, and 5. Figure 11b shows the traces of the GBs of grains 3, 4, and 5 showing their geometric arrangement in 2D. The out of (film) plane slip planes of grains 4 and 5 are almost mutually orthogonal to the slip planes of grain 3. Somewhat similar, the traces of the {111} slip planes of grains 4 and 6 are also at high angles to each other (Figure S4). The (almost) orthogonal sets of slip planes constitute geometrically highly incompatible systems for slip transmission, leading to dislocation pile-ups at these GBs (Figure S5). During deformation, these dislocation pile-ups at the GBs will result in a stress gradient developing across the GBs. Only when a sufficiently high stress level has been reached at the GB should dislocations nucleate into the neighboring grain [61]. In a series of grains connected by such incompatible grain boundaries, this would mean that one grain is rotating due to dislocation motion, and after a certain rotation (stress buildup), the rotation of a neighboring grain would be initiated, which would afterwards drive the rotation in the next connected grain (e.g., grains 3–4 and 4–6 in Figure 4 and Figure 7). From these observations, we propose that the geometric incompatibility of the slip systems across the grain boundaries plays a critical role in the CGR.

The strongest evidence of dislocation pile-up formation is seen in grain 11, as the specimen is unloaded to ε = 5.1% (Figure 10d), and the GND density is mostly concentrated in the vicinity of the GBs, especially at the boundary between grains 3 and 11 (as indicated by an arrow in Figure 10d). As the specimen is further unloaded to ε = 3.6% (Figure 10e), the GND density in grain 11 abruptly reduces and the density within grain 3 significantly increases. Thus, the back stresses that developed at GB 3–11 during the unloading would be so severe that the dislocations propagate across grain 3, driving the rotation. A similar effect is observed for grain pairs 9 and 10 during unloading from ε = 5.1% to ε = 3.6% (Figure 10d,e), where the density reduces at the GB (GB 9–10) in grain 9 and increases in grain 10 (as indicated by an arrow in Figure 10d,e), thus driving the CGR for these pairs as well, which could be seen in the STEM series (Figure 6f).

## 4. Conclusions

In summary, we demonstrated a cooperative and reversible rotation of grains in nc Pd thin films accompanied by a strong BE using in situ cyclic loading–unloading experiments combining μp-STEM imaging and quantitative ACOM-STEM. A careful local analysis was performed based on the Nye tensor approach to determine the scalar GND density. The evolution of the GND density within the grains showed evidence of dislocation activity contributing to the lattice rotation. The GND density in a selected set of neighboring grains showed a significantly different behavior due to the heterogeneous stress field as the specimen was loaded and unloaded. Further, based on the GND analysis, dislocation pile-up formation was observed at the GBs as the specimen was unloaded. The dislocation pile-up formation at the GBs clearly showed the generation of back stresses during the unloading of the specimen that led to the BE. The role of the geometric incompatibility of the slip systems across GBs was invoked to explain the dislocation pile-up formation at the GBs. Only after sufficient stress buildup that dislocations are transmitted into a neighboring grain and induce rotation of this grain. This process can continue over multiple grains, leading to a stepwise cooperative grain rotation, and is (partially) reversible during unloading. To rationalize this, the abrupt reduction of the GND density at the GBs on unloading was seen in conjunction with the enhancement of the GND densities in the neighboring grain, which showed the CGR. The lack of texture development during loading and unloading revealed grain rotation, which, to a large extent, does not depend on the orientation of the grain with respect to the global straining direction. This fits to both GB-mediated processes and dislocation processes in the presence of a strong local stress buildup. Therefore, from our in situ TEM experimental approach, we have been able to provide greater insights into the cyclic behavior of nc metals exhibiting a strong BE, which is associated with a strong local stress development leading to complex dislocation-based interactions between different grains that lead to the cooperative grain rotations.

## Data Availability

The data presented in this study are available on request from the corresponding author.

## Supplementary Materials

The following are available online at https://www.mdpi.com/2079-4991/11/2/432/s1, Figure S1: Bright-field TEM images of nc Pd (a) as sputtered and aged for 18 months (b) after in situ annealing of the structure in (a). Figure S2: Secondary scanning electron microscopy images of (a) sputtered and heat-treated Pd thin film lifted using a transfer frame, (b) the PTP device as seen from the side with a 52° stage tilt. The red-dotted box indicates the area where the thin film is transferred. (c) The final sample with the region of interest shown using the red-dotted box. Figure S3: (a) Grain rotation maps evaluated for the entire ACOM-TEM series during loading, unloading, and reloading and the corresponding strain (in %). The colored grains in each map are the ones analyzed, and their rotation (in degrees) is colored as indicated by the color bar. The grains that are not colored were not analyzed. The red-dotted square encompassed the rotation maps, indicating bending of the sample around an axis of approximately 45° to the loading direction due to an elongation of the film during the earlier straining experiments followed in BFTEM. These maps were excluded from a detailed data analysis. (b) Grain rotation map series excluding the bend states indicated in (a) with a 3.6% strain map as a reference state. No long-range rotation gradient is visible, indicating that essentially no bending/tilting of the sample occurred during this part of the straining series. The blue-dotted box shows the region from which the grains were selected for the detailed analysis. They are numbered as in Figure 2b. Figure S4: Traces of {111} planes of grains 4 and 6. Other marking conventions are the same as shown in Figure 8. (b) Grains 4 and 6 with the traces of their grain boundaries. Figure S5: Schematic showing the geometric incompatibility of slip systems for grains 3, 4, and 6 with their slip planes arranged almost orthogonal to each other, leading to dislocation pile-up at the GBs. Table S1: Schmid factors for in-plane and out-of-plane slip components for grains 1 to 10 as marked in Figure 2b. Table S2: Misorientation axis and angle for the selected pairs of grains before and after deformation and the angular deviation in specimen reference. Movie S1: BFTEM image during loading and unloading showing back-and-forth motion of dislocation pointed by an arrow. Movie S2: Microprobe STEM image series showing cooperative rotation of grains as illustrated in Figure 6.
