# Supplementary material for: In Situ TEM Observation of Cooperative Grain Rotations and the Bauschinger Effect in Nanocrystalline Palladium
Source: Nanomaterials (Basel). 2021 Feb 9;11(2):432. doi: 10.3390/nano11020432 (PMC7915181; doi:10.3390/nano11020432)
Supplement: Supplementary file 1 [file nanomaterials-11-00432-s001.zip › nanomaterials-1086570-supplementary/nanomaterials-1086570-supplementary.pdf]

nanomaterials

Supporting Information

In Situ TEM Observation of Cooperative Grain Rotations and the Bauschinger Effect in Nanocrystalline Palladium

Ankush Kashiwar[1,2,4,†], Horst Hahn[1,2] and Christian Kübel[1,2,3,*]

1 Institute of Nanotechnology, Karlsruhe Institute of Technology, Hermann-von-Helmholtz Platz 1, 76344 Eggenstein-Leopoldshafen, Germany; ankush.kashiwar@partner.kit.edu (A.K.); horst.hahn@kit.edu (H.H.)
2 Department of Materials and Earth Sciences, KIT-TUD Joint Research Laboratory Nanomaterials, Technische Universität Darmstadt, Alarich-Weiss-Straße 2, 64287 Darmstadt, Germany
3 Karlsruhe Nano Micro Facility (KNMF), Karlsruhe Institute of Technology, Hermann-von-Helmholtz Platz 1, 76344 Eggenstein-Leopoldshafen, Germany
4 Institute of Mechanics, Materials and Civil Engineering, Université catholique de Louvain, Place Sainte Barbe 2, B-1348 Louvain-la-Neuve, Belgium
* Correspondence: christian.kuebel@kit.edu (C.K.); Tel.: +49 721 608 28970 (C.K.)
† Present address: Electron Microscopy for Materials Science (EMAT), University of Antwerp, Groenenborgerlaan 171, B-2020 Antwerp, Belgium

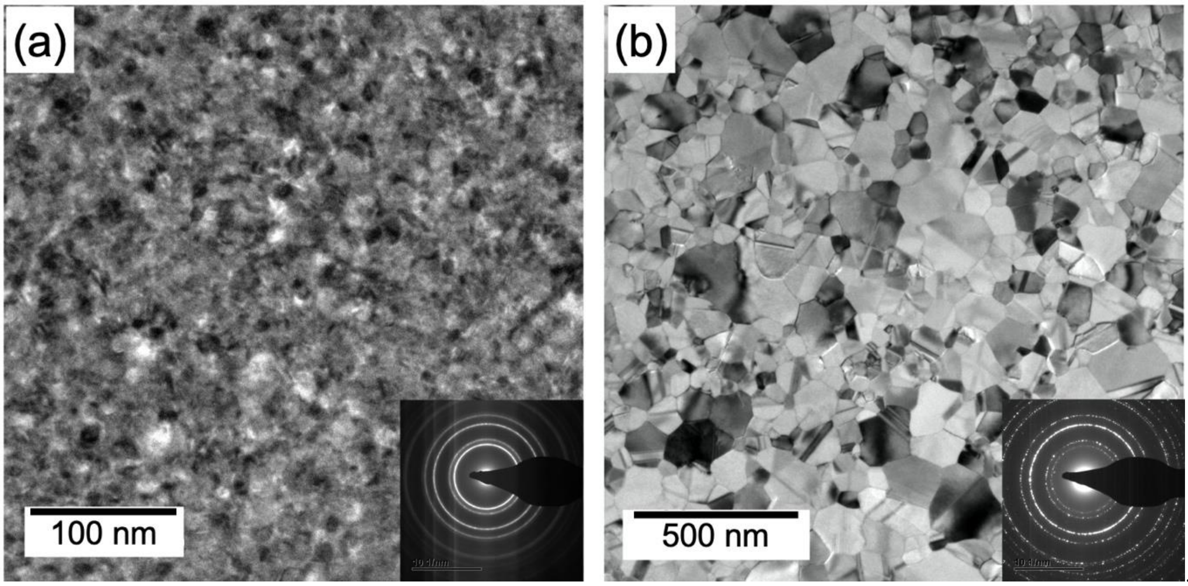

Figure S1. Bright-field TEM images of nc Pd (a) as sputtered and aged for 18 months (**b**) after *in situ* annealing of the structure in (**a**).

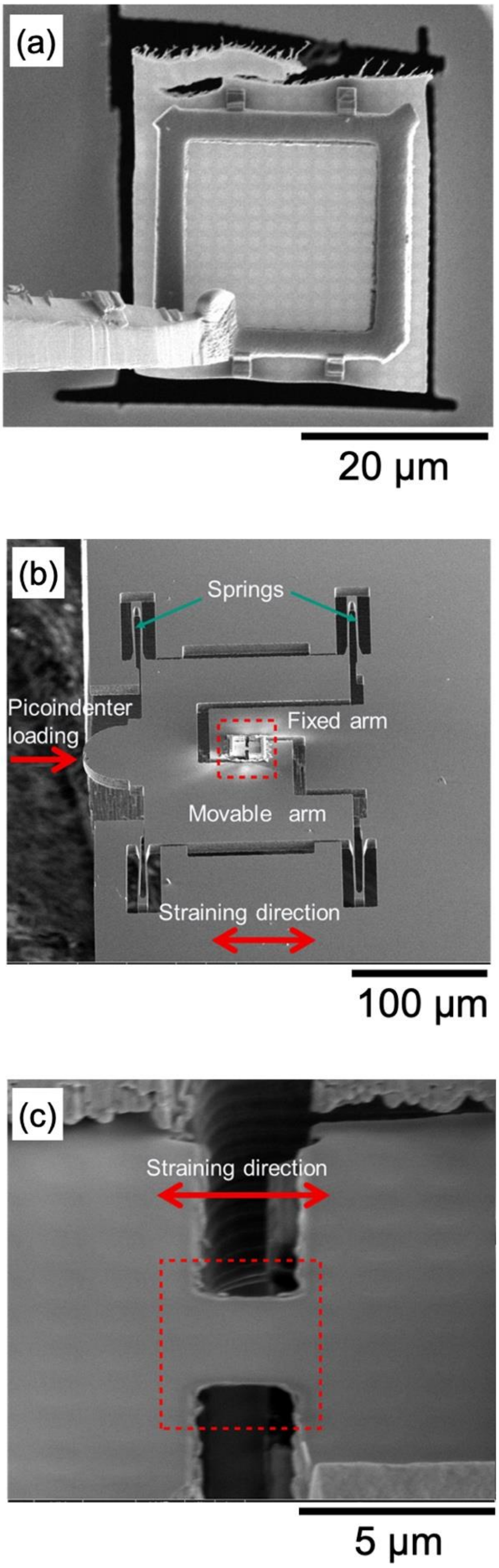

Figure S2. Secondary electron scanning electron microscopy images of (**a**) sputtered and heat-treated Pd thin film lifted using a transfer frame, (**b**) the PTP device as seen from side with 52° stage tilt, red dotted box indicates the area where the thin film is transferred, (**c**) the final sample with the region of interest is shown using red dotted box.

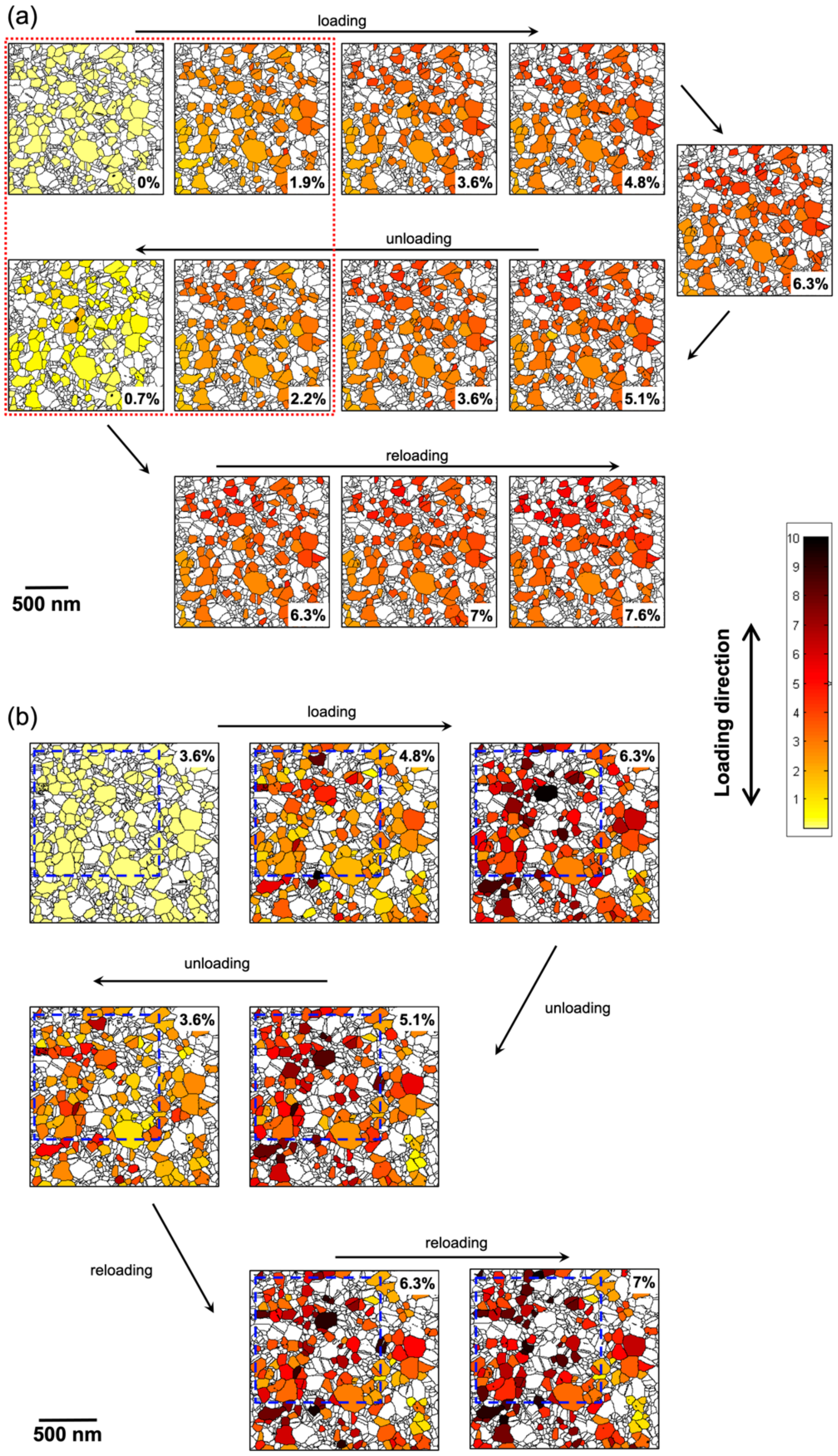

Figure S3. (**a**) Grain rotation maps evaluated for the entire ACOM-TEM series during loading, unloading and re-loading and the corresponding strain (in %). The colored grains in each map are the ones analyzed and their rotation (in degrees) is colored as indicated by the color bar. The grains that are not colored were not analyzed. The red-dotted square encompassed the rotation maps indicating bending of the sample around an axis approximately 45° to the loading direction due to an elongation of the film during the earlier straining experiments followed in BF-TEM. These maps were excluded from the detailed data analysis. (**b**) Grain rotation map series excluding the bend states indicated in (a) with the 3.6% strain map as a reference state. No long-range rotation gradient is visible, indicating that essentially no bending/tilting of the sample occurred during this part of the straining series. The blue dotted-box shows the region from which the grains were selected for the detailed analysis. They are numbered as in Figure 2b.

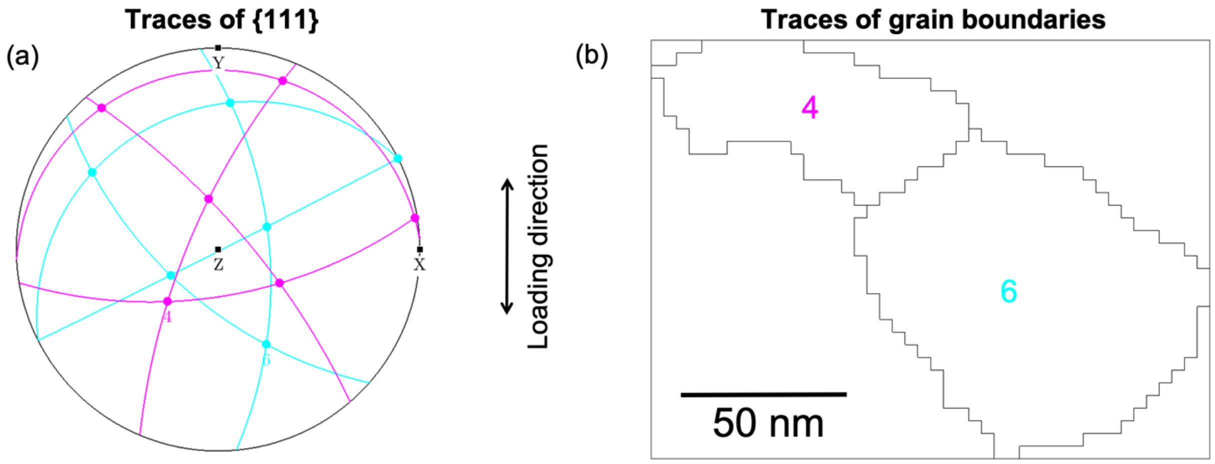

Figure S4. (a) Traces of {111} planes of grains 4 and 6, other marking conventions are same as shown in Figure 8 and (b) grains 4 and 6 with the traces of their grain boundaries.

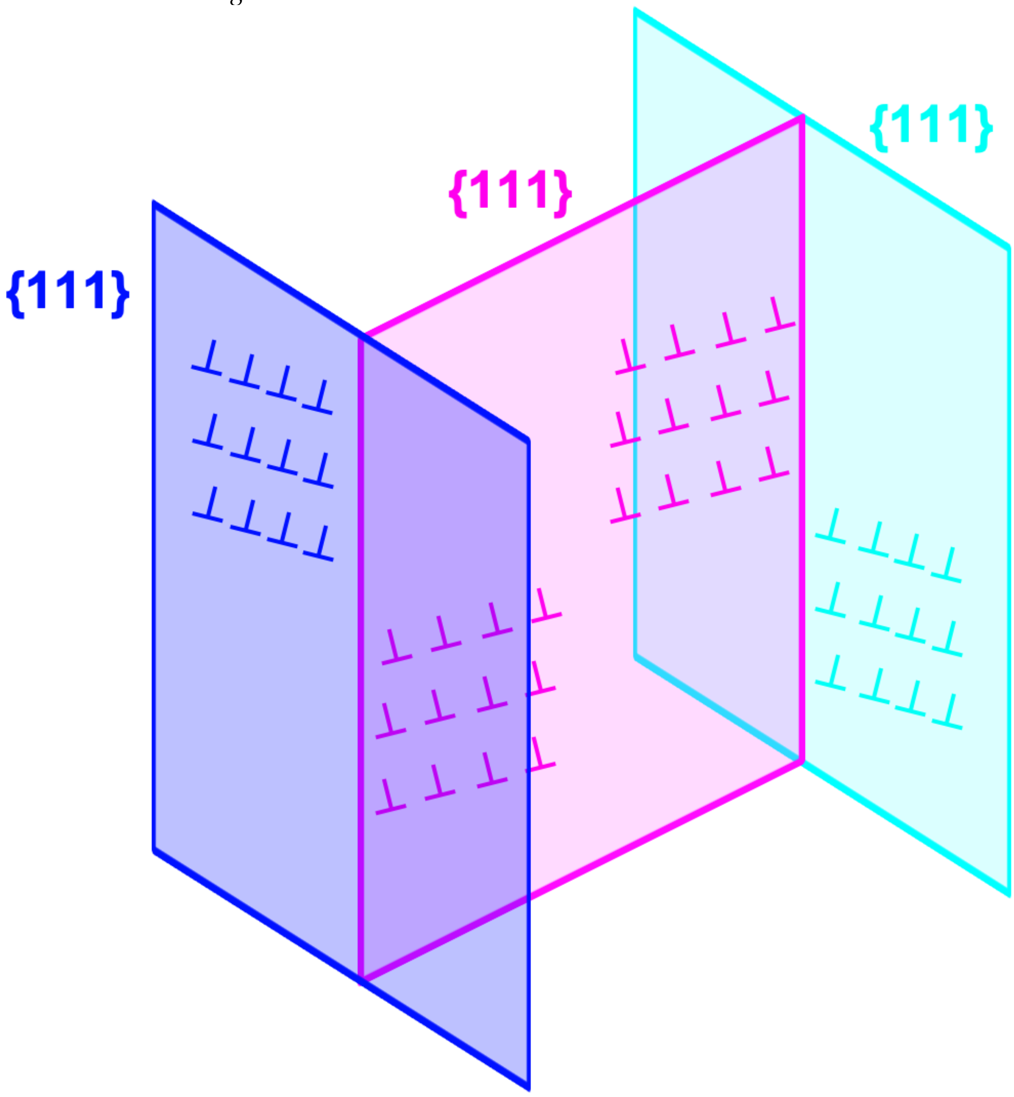

Figure S5. Schematic showing the geometric incompatibility of slip systems for grains 3, 4 and 6 with their slip planes arranged almost orthogonal to each other leading to dislocation pile-up at the GBs.

Table S1. Schmid factors for in-plane and out-of-plane slip components for grains 1 to 10 as marked in Figure 2b.

Slip components	Slip directions	Slip planes	Schmid factor (Grain ID)									
	<uvw>	{hkl}	1	2	3	4	5	6	7	8	9	10
In- plane	[0 -1 1]	(1 1 1)	0.097	0.057	0.05	0.11	0.09	0.15	0.07	0.04	0.006	0.007
	[1 0 -1]	(1 1 1)	0.13	0.113	0.08	0.02	0.02	0.29	0.013	0.014	0.05	0.056
	[-1 1 0]	(1 1 1)	0.04	0.055	0.03	0.09	0.07	0.14	0.09	0.059	0.05	0.05
Out-of- plane	[-1 1 0]	(1 1 -1)	0.27	0.417	0.33	0.48	0.49	0.4	0.35	0.28	0.34	0.46
	[-1 0 -1]	(1 1 -1)	0.005	0.07	0.01	0.29	0.32	0.21	0.18	0.15	0.1	0.16
	[0 -1 -1]	(1 1 -1)	0.28	0.34	0.34	0.18	0.16	0.19	0.17	0.13	0.23	0.3
	[0 -1 1]	(-1 1 1)	0.42	0.38	0.43	0.34	0.28	0.32	0.48	0.48	0.08	0.1
	[-1 0 -1]	(-1 1 1)	0.003	0.06	0.009	0.17	0.14	0.16	0.29	0.33	0.26	0.26
	[-1 -1 0]	(-1 1 1)	0.42	0.45	0.43	0.17	0.14	0.48	0.18	0.14	0.18	0.36
	[1 0 -1]	(1 -1 1)	0.14	0.02	0.1	0.14	0.2	0.07	0.12	0.19	0.42	0.36
	[-1 -1 0]	(1 -1 1)	0.1	0.01	0.06	0.4	0.42	0.05	0.26	0.19	0.09	0.15
	[0 -1 -1]	(1 -1 1)	0.04	0.011	0.04	0.26	0.21	0.016	0.39	0.39	0.32	0.21

Table S2. Misorientation axis and angle for the selected pairs of grains before and after deformation and the angular deviation in specimen reference.

Grain pairs ID	Misorientation axis-angle pairs				Angle between rotation axes
	Initial (i) (ε = 3.6 %)		Final (f) (ε = 4.8 %)		
	Axis	Angle	Axis (f) ∠ (i)	Angle	
**1-2**	[-3 7 11] (i)	20.1°	[-3 7 11] (f) ∠1.9° [-3 7 11] (i)	19.9°	61°
**2-3**	[-7 4 12] (i)	15.4°	[-7 4 12] (f) ∠0.2° [-7 4 12] (i)	14.8°	83.5°
**3-4**	[-8 5 10] (i)	31.1°	[-8 5 10] (f) ∠0.8° [-8 5 10] (i)	30.9°	79°
**3-5**	[-9 7 12] (i)	26.4°	[-9 7 11] (f) ∠3° [-9 7 12] (i)	25.6°	17°
**4-5**	[-1 10 12] (i)	5°	[-1 5 9] (f) ∠10.8° [-1 10 12] (i)	5.6°	65.5°
**5-6**	[-3 7 9] (i)	50°	[-3 6 8] (f) ∠1.1° [-3 7 9] (i)	50.6°	60.2°
**4-6**	[-2 4 5] (i)	45.1°	[-2 4 5] (f) ∠0.2° [-2 4 5] (i)	45.1°	57.7°
**6-8**	[-1 0 3] (i)	30.8°	[-1 0 3] (f) ∠0.3° [-1 0 3] (i)	30.7°	45°
**5-7**	[-5 11 12] (i)	21.7°	[-11 5 11] (f) ∠2.9° [-5 11 12] (i)	21.7°	81.5°
**9-10**	[-11 11 12] (i)	58.6°	[-12 11 12] (f) ∠0.2° [-11 11 12] (i)	59°	50°
**7-9**	[-11 11 12] (i)	46.9°	[-10 10 11] (f) ∠0.3° [-11 11 12] (i)	47.2°	84.3°

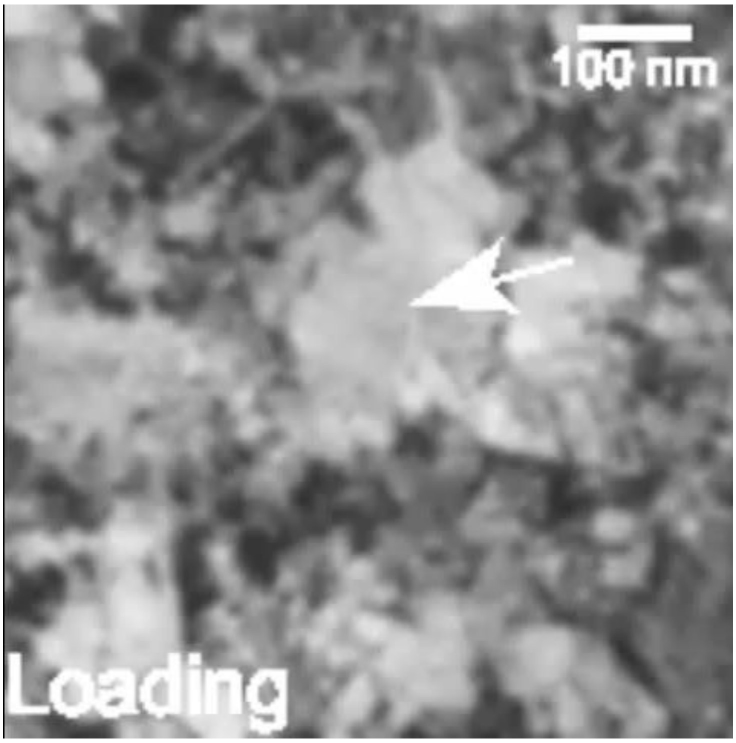

Movie S1. BFTEM image during loading and unloading showing back and forth motion of dislocation pointed by arrow.

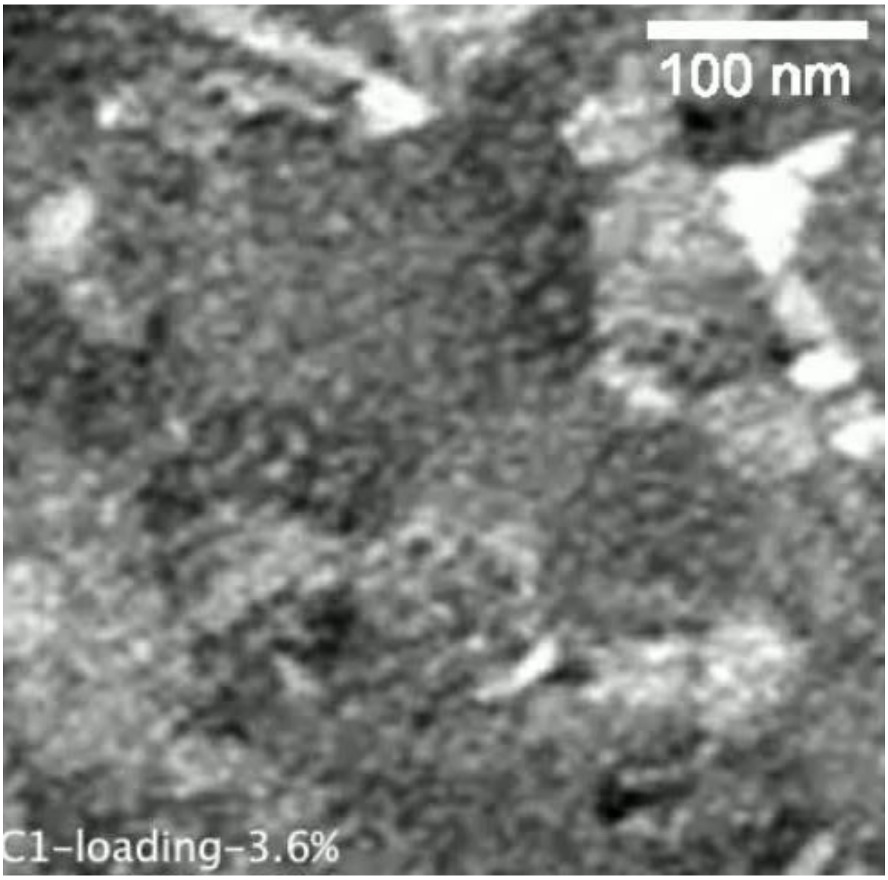

Movie S2. Microprobe STEM image series showing cooperative rotation of grains as illustrated in Figure 7.
